# *Inonotus obliquus* polysaccharide are linear molecules that alter the abundance and composition of intestinal microbiota in Sprague Dawley rats

**DOI:** 10.3389/fnut.2023.1231485

**Published:** 2023-09-28

**Authors:** Songqing Liu, Wenjing Zhou, Xin Deng, Wei Jiang, Yanping Wang, Jiasui Zhan, Binhong Hu

**Affiliations:** ^1^College of Chemistry and Life Science, Chengdu Normal University, Chengdu, China; ^2^Sichuan Provincial Key Laboratory for Development and Utilization of Characteristic Horticultural Biological Resources, Chengdu Normal University, Chengdu, China; ^3^College of Veterinary Medicine (Institute of Comparative Medicine), Yangzhou University, Yangzhou, China; ^4^Department of Forest Mycology and Plant Pathology, Swedish University of Agricultural Sciences, Uppsala, Sweden

**Keywords:** *Inonotus obliquus* polysaccharide, structure of IOP, gut microbiota, gender differences, rats

## Abstract

**Introduction:**

The macromolecular polysaccharide *Inonotus obliquus* polysaccharide (IOP) is composed of various monosaccharides, and it could modulate the composition and diversity of intestinal flora. However, its impact on the intestinal flora in rats of different genders remains unclear. Therefore, this study aims to investigate the structural changes of IOP and its effects on the intestinal flora after administration in male and female rats.

**Methods:**

In this study, the molecular weight and purity of IOP were analyzed by high-performance gel permeation chromatography (HPGPC) and phenol sulfuric acid method, and NMR was used to confirm the chemical structure of IOP. Sex hormone [testosterone (T) and estradiol (E2)] levels and intestinal microbial changes were detected by enzyme-linked immunosorbent assay (ELISA) and 16S rRNA, respectively, after gavage of IOP (100 mg/kg) in male and female Sprague Dawley (SD) rats.

**Results:**

HPGPC analysis showed that the average molecular weight (Mw) of IOP was 4,828  Da, and the total sugar content of the purified IOP was 96.2%, indicating that the polysaccharide is of high purity. NMR revealed that IOP is a linear macromolecule with an α-D-type glucose backbone. The results of ELISA and 16S rRNA showed that the IOP increased the abundance of beneficial bacteria, such as *Clostridia_UCG-014* and *Prevotellaceae_NK3B31*, and reduced that of harmful bacteria, such as *Colidextribacter* and *Desulfobacterota* in the intestine of both male and female rats, and IOP changed the levels of sex hormones in male and female rats. Further analyses revealed that the increase in alpha diversity was higher in male than female rats. α diversity and β diversity revealed a significant difference in the composition of cecal microbiota between male and female rats in the control group, but IOP intake reduced this difference. Meanwhile, α analysis revealed a change in the composition of bacterial flora was more stable in male than female rats.

**Conclusions:**

This study enhances our comprehension of the IOP structure and elucidates the alterations in intestinal flora following IOP administration in rats of varying genders. Nonetheless, further investigation is warranted to explore the specific underlying reasons for these discrepancies.

## 1. Introduction

Plant polysaccharides are ubiquitous biological macromolecules that have garnered increasing attention for their unique bioactivities and wide range of applications in anti-tumor, anti-inflammatory, antibacterial, and hypoglycemic research (1). Some of the plants that secrete notable polysaccharides include *Astragalus membranaceus* (2), *Lycium barbarum* (3), and *pumpkin* (polysaccharide) (4, 5), among others. To date, nearly 100 plant-derived polysaccharides have been isolated and purified. Specific dosages of water-soluble plant polysaccharides have diverse biological functions, including enhancing animal growth performance and intestinal environment (6, 7). Plant polysaccharides do not have toxic residues that could accumulate in human or livestock bodies and do not contribute to the development of drug resistance. Therefore, they are widely utilized as additives either alone or in feeds. *Inonotus obliquus*, a fungus that thrives well in the sub frigid zone, exhibits diverse biological activities and has been applied extensively in the pharmaceutical and health product industries (8, 9). Although our previous HPLC analysis identified the major components of IOP, including Mannose (Man), Glucuronic acid (Glu-A), Glucose (Glu), Galactose (Gal), Xylose (Xyl), Arabinose (Ara) and Fucose (Fuc) (10), the unique structural features of IOP remain unknown.

The gastrointestinal tract is a complex ecosystem in which the abundance and composition of microbiota vary with gut site, and they play different functions (11). The gut microbiota is a dynamic ecosystem that plays a crucial role in the digestion of food components and the host’s immune response (12). Complex polysaccharides serve as the primary carbon source for anaerobic bacteria in the posterior gut (cecum and colorectum). Members of *Bacteroidaceae* and *Ruminococcaceae* family can break down these complex plant polysaccharides (13–15). *Firmicutes* and *Bacteroidota*, present in the gut, have been linked with the development of several diseases, the most common being obesity (16, 17). The composition of gut microbiota is also affected by various factors, such as medication (18), diet (19, 20), age (21), and gender (22). However, the difference between sexes is the first factor affecting the intestinal barrier, the intestinal permeability of women is generally lower than that of men, and this may be related to the secretion of estrogen (23, 24). Increasing evidence has shown that the development of many diseases is closely related to sex, and certain differences exist in the composition of intestinal flora between sexes of different species (25, 26). The composition and diversity of gut microbiota also affect the secretion of sex hormones. For example, β-estradiol directly affects the secretion of inflammatory factors (IL-12, etc.) secreted by immune cells, which alters intestinal permeability by promoting inflammatory response and altering the intestinal flora abundance and composition of intestinal flora. Gut microbiota also plays an important role in the metabolism of testosterone (27–29). Therefore, some scholars believe that sex hormones may be the primary factor affecting the composition of intestinal flora between genders (22).

This study aimed to analyze the IOP structure using high-performance gel permeation chromatography (HPGPC) and nuclear magnetic resonance (NMR). After intragastric administration of IOP to male and female SD rats, *16S rRNA* sequencing technology was used to analyze the differences in the cecal flora between the sexes.

## 2. Materials and methods

### 2.1. The extraction of *Inonotus obliquus* polysaccharide

IOP was extracted as previously described (10). Briefly, *Inonotus obliquus* (IO) was crushed, and fat was removed using petroleum ether, followed by traditional hot water extraction (4, 30). The above process was repeated three times before adding 1% trichloroacetic acid for deproteinization. The extract was then collected and concentrated, and three volumes of absolute ethanol were added, followed by overnight incubation at 0°C. The precipitate was centrifuged to obtain the crude extract, which was washed 2–3 times with absolute ethanol. To purify the crude polysaccharide, first, DEAE-52 cellulose was soaked in 0.5 mol/L hydrochloric acid for 1 h. Then, impurities in the resin were washed away bay 0.5 mol/L hydrochloric acid, and the resin was eluted with distilled water, 4–5 times its volume, until it reached a neutral pH. Finally, the resin was buffer with distilled water at a flow rate of 5 mL/min for 2 h. Afterwards, the sample was prepared for loading. The crude polysaccharide was dissolved in distilled water, heated, and shaken. The mixture was centrifuged at 12,000 rpm for 2 min, and the supernatant was collected for loading. Subsequently, 2% sodium nitrite solution was added to the polysaccharides at a ratio of 1:2 (polysaccharides: 2% sodium nitrite solution), and the mixture was heated in a water bath at 80°C. Activated carbon was added, and the mixture was stirred, heated for 1 h, and incubated at 4°C overnight. Finally, the supernatant was centrifuged, and the solution was freeze-dried to obtain IOP.

### 2.2. Determination of total sugar content in *Inonotus obliquus* polysaccharide

First, a standard curve was plotted. Here, 1 mg of standard dextran (Yuanye Bio-Technology Co., Ltd., Shanghai, China) was weighed into a 1 mL volumetric flask. The flask was then topped up to 1 litre. Standard dextran solutions of 8 mg/mL, 4 mg/mL, 2 mg/mL, 1 mg/mL, 0.5 mg/mL, 0.25 mg/mL, and 0.125 mg/mL, each 1 mL, were then prepared. Thereafter, 200 μL of 6% phenol (Komio Chemical Reagents Co., Ltd., Tianjin, China) and 1 mL of concentrated sulfuric acid (Hushi Laboratorial Equipment Co., Ltd., Shanghai, China) were added. The mixture was left to stand for 10 min, shaken well, and allowed to stand further at room temperature for 20 min. The absorbance was measured at 490 nm. Distilled water used as a blank and the same colorimetric procedure was followed. A standard curve of polysaccharide concentration (*x*-axis) and absorbance (*y*-axis) was then plotted.

The sample content was then determined. Distilled water (200 μL) was added into 1 mg of IOP obtained in section 2.1. The absorbance was measured in triplicate as described in the preceding section. The polysaccharide content was calculated using the standard curve.

### 2.3. Determination of molecular weight of *Inonotus obliquus* polysaccharide

The molecular weight of IOP was determined using the high-performance gel permeation chromatography (HPGPC) method. First, the mobile phase solution was prepared by dissolving 11.688 g of NaCl in purified water and transferring it to a 1 L volumetric flask. After sonication for 10 min, the mixture was filtered through a 0.22 μm pore membrane. The standard solution was then prepared. Eight dextran standard samples (2 mg each) were weighed and dissolved in 1 mL of the mobile phase solution to prepare a 2 mg/mL solution. The solution was transferred to 1.8 mL sample vials. The sample (2 mg) was dissolved in 1 mL of the mobile phase solution, sonicated for 10 min, and centrifuged at 12000 rpm for 10 min. The supernatant was filtered through a 0.22 μm membrane filter and transferred to a 1.8 mL sample vial. Finally, the samples were then analyzed. In summary, the standard samples were placed in the sample tray for chromatographic analysis under the following experimental conditions; mobile phase: 0.2 M NaCl solution; chromatographic column: BRT105-103-101 gel permeation column (8× 300 mm); flow rate: 0.8 mL/min; column temperature: 40°C; injection volume: 25 μL; detector: refractive index detector (RID-10A); analysis time: 60 min. The retention time was obtained and the standard curves of lgMp-RT (Mp peak molecular weight), lgMw-RT (Mw average molecular weight), and lgMn-RT (Mn number-average molecular weight) were plotted. The retention time of the sample was used to calculate the molecular weight (Mp, Mw, and Mn).

### 2.4. Nuclear magnetic resonance spectroscopy

Here, 600ul of D_2_O reagent was added to 100 mg IOP in a Nuclear Magnetic Resonance (NMR) tube. After dissolving the IOP, ID (^1^H and ^13^C) and 2D (HSQC, HMBC, COSY) spectra were measured using a 600 MHz Bruker (Germany). A zg30 pulse sequence was used for ^1^H, and the sample was scanned 32 times. A zgpg30 pulse sequence was used at ^13^C, and the test scans were performed 1,024 times. The Cosygpppqf, noesygpphpp and Hsqcedetgp pulse sequences were used for the two-dimensional spectra, and four test scans were performed.

### 2.5. *In vitro* cell experiment

LS411N cells were cultured to prepare a single cells’ suspension. After culturing, the culture medium was poured out, and the residual culture medium was washed using PBS. The cell monolayer was separated into single cells using 0.25% trypsin for 1 min. The digestion was terminated by adding a culture medium containing 10% FBS. The cell suspension was poured into a 15 mL centrifuge tube and centrifuged at 300–400 × g for 5 min at 4°C. The supernatant was discarded, and the cell pellet was washed two times with PBS through centrifugation at 300–400 × g for 5 min at room temperature. Then the cells were resuspended in flow loading buffer for counting and viability determination. The cell density was adjusted to 1 × 10^7^ cells /mL.

Flow cytometry experiment was then performed. First, 1–3 × 10^6^ cells were collected, washed twice with precooled PBS, centrifuged at 300 g for 5 min (model DM0412S, SCILOGEX, United States), and the supernatant was discarded. A few cells were picked from the sample tube and one blank tube to set up two single-staining tubes. Cells were washed once with precooled PBS, the supernatant was discarded after centrifugation, and the cells were resuspended in 500 μL of precooled 1× Binding Buffer working solution. Next, 5 μL of Annexin V-FITC (United Biology, Hangzhou, China) and 10 μL of PI staining solution were added to each sample tube, and 5 μL Annexin V-FITC or 10 μL PI was added to the two single-staining tubes, vortexed gently, mixed, and incubated at room temperature in the dark for 5 min. Flow cytometric assessment was then performed (model CytoFLEX, Beckman coulter, United States), and the voltage of FSC, SSC, FITC and PI channels was adjusted using a blank tube. Under this voltage, the compensation between FITC and PI was adjusted with a single staining tube before loading the sample. IOP of 100 μg/mL and 200 μg/mL were used to detect the apoptosis rate experiment.

### 2.6. *In vivo* experiment

Forty-eight Sprague Dawley (SD) rats (female: 150 ± 10 g, male: 180 ± 10 g) were purchased from the Chengdu Dashuo Experimental Animal Co., Ltd. (Chengdu, China). The rats were housed under constant conditions (temperature 25 ± 3°C, humidity 75 ± 5%), were provided with enough food and water, and were reared at 12 h light and 12 h darkness cycle. All laboratory procedures, including those related to animal handling, welfare and euthanasia, were carried out in accordance with the guidelines and regulations of ARRIVE, and the protocol for the animal studies study was approved by the Animal Care Office of Chengdu Normal University (No: CDNU-2021092614 M). After 7 days of acclimation, the mice were randomly divided into the following four groups (*n* = 12): control-female group, control-male group, IOP-female group and IOP-male group. The IOP group received 100 mg/kg by gavage (31), while the control group received the same proportion of normal saline. After 4 weeks of treatment, the rats were anaesthetized with ether and sacrificed by neck dislocation. Cecal contents were collected in a sterile environment and stored in a refrigerator at −80°C after quick freezing in liquid nitrogen.

### 2.7. Measurement of sex hormone levels

Whole blood was centrifuged at 3000 g for 10 min to obtain serum. Serum testosterone (T) and estradiol (E2) levels were measured using an ELISA kit (Hepeng Biotechnology Co., Ltd., Shanghai, China), according to the manufacturer’s instructions.

### 2.8. High-throughput sequencing of the *16S rRNA*

#### 2.8.1. DNA extraction and amplification

Caecal content samples were snap frozen and stored at −80°C. Bacterial DNA was extracted from the caecal contents using a DNeasy PowerSoil kit (Qiagen, Hilden, Germany) following the manufacturer’s instructions. DNA concentration and integrity were measured by a NanoDrop 2000 spectrophotometer (Thermo Fisher Scientific, Waltham, MA, United States) and agarose gel electrophoresis, respectively. PCR amplification of the V3-V4 hypervariable regions of the bacterial *16S rRNA* gene was carried out in a 25 μL reaction volume using universal primer pairs (343F-5′-TACGGRAGGCAGCAG-3′, 798R-5′-AGGGTATCTAAT CCT-3′). The reverse primer contained a sample barcode, and both primers were connected with an Illumina sequencing adapter.

#### 2.8.2. Library construction and sequencing

The Amplicon quality was visualized using gel electrophoresis. The PCR products were purified with Agencourt AMPure XP beads (Beckman Coulter Co., United States) and quantified using the Qubit dsDNA assay kit. The DNA concentrations were then adjusted for sequencing. Sequencing was performed using the Illumina NovaSeq6000 platform with two paired-end read cycles of 250 bases each (Illumina Inc., San Diego, CA; OE Biotech Company, Shanghai, China).

#### 2.8.3. Bioinformatics analysis

Raw sequencing data were in FASTQ format. Paired-end reads were then preprocessed using the cut adapt software to detect and cut off the adapter. The chimera reads were cut off using DADA2 (32) under default QIIME parameters (33). In the end, representative reads, and the ASV abundance were generated. The representative read of each ASV was selected using QIIME 2 package. All representative reads were annotated and blasted against data in the Silva database Version 138 (or unite) (*16S rDNA*) using q2-feature-classifier under default parameters. The microbial diversity in caecal content samples was estimated using the alpha diversity that includes Simpson and Shannon indices. The Bray Curtis distance matrix performed by QIIME software was used for Bray Curtis Principal coordinates analysis (PCoA) and phylogenetic tree construction. Sequencing of the *16S rRNA* gene was performed by OE Biotech Co., Ltd. (Shanghai, China).

### 2.9. Statistical analysis

Numerical results of normally distributed variables were expressed as mean ± standard deviation. Data were analyzed using IBM SPSS Statistics v26.0 and GraphPad Prism 8 (GraphPad InStat Software, United States) software. α diversity analysis was analyzed using *t*-test, the linear discriminant analysis effect size (LEfSe) analysis initially employed the Kruskal–Wallis rank sum test to identify species exhibiting significant differences in abundance across different groups. Subsequently, the Wilcoxon rank sum test was utilized to assess intergroup disparities. Finally, linear discriminant analysis (LDA) was applied for data reduction and evaluation of the impact of significantly distinct species (LDA score). The remaining analyses were conducted using one-way analysis of variance (ANOVA). A significance level of *p* < 0.05 was considered statistically significant.

## 3. Result

### 3.1. Elution curve and total sugar content of *Inonotus obliquus* polysaccharide

**Figure 1:**
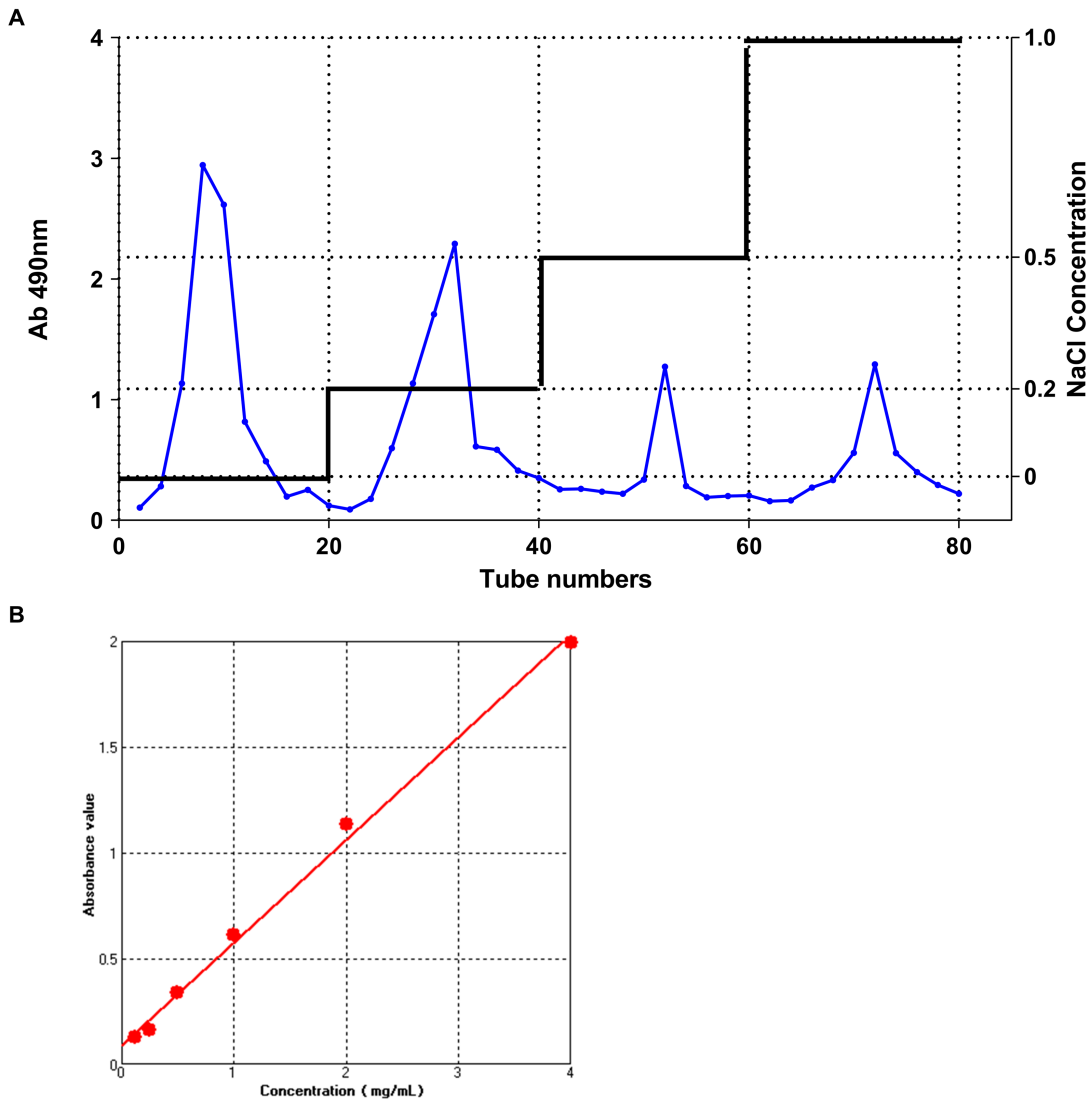
The DEAE-52 cellulose column elution curve and Dextran standard curve. **(A)** The DEAE-52 cellulose column elution curve of IOP. From left to right; the peaks correspond to the following eluents: M-water, M-0.2 M NaCl, M-0.5 M NaCl, and M-1.0 M NaCl. **(B)** Standard curve for measuring glucose concentration using the phenol-sulfuric acid method.

Crude polysaccharides were purified and a gradient elution curve was obtained according to the method described in section 2.1 (Figure 1A). Four distinct elution peaks which correspond to elution with distilled water, 0.2 M NaCl, 0.5 M NaCl, and 1.0 M NaCl solutions were obtained. Elution with distilled water generated the highest polysaccharide content. Therefore, the polysaccharide fraction obtained from the first elution with distilled water was collected, freeze-dried, and used in subsequent analyses.

The total sugar content of IOP is shown in Figure 1B. The regression equation for the relationship between absorbance and polysaccharide concentration was *y* = 0.09021 + 0.48593*x* (where *x* represents the concentration of dextran in mg/mL and *y* represents the absorbance value at 490 nm for various concentrations of glucose). The coefficient of determination (*R*^2^) was 0.99572. By substituting the absorbance values of IOP measured at 490 nm into the dextran standard equation, the total sugar content of IOP was determined to be 96.2%, implying that a highly pure IOP was obtained.

**Figure 2:**
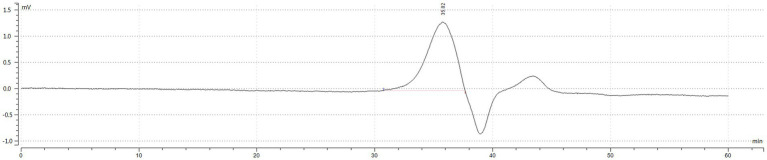
HPGPC outflow curve diagram. The vertical axis (MV) represents the difference in refractive index between the sample and the solvent, and the response signal of the differential refractive index detector is directly proportional to the solute concentration. The horizontal axis represents the retention time. According to the principle of high-performance gel permeation chromatography (HPGPC), the elution of compounds decreases with molecular weight. The main polymer elution peak occurred at 35.823 min, and the peak at 39.0 min is the peak of the mobile phase, and the peak after the mobile phase is the salt or oligosaccharide contained in the IOP.

### 3.2. Molecular weight determination of *Inonotus obliquus* polysaccharide

As shown in Table 1 and Figure 2 the peak molecular weight (Mp) at the peak apex was 4,489 Da. The average molecular weight (Mn), calculated by considering the number of molecules weighted by their abundance, was 4,549 Da. The average molecular weight (Mw), which was calculated by considering the mass of the molecules weighted by their abundance, was 4,828 Da. The polydispersity coefficient (PD), which represents the dispersion of the substance, and is the ratio of Mw to Mn (PD = Mw/Mn), was 1.061. Generally, a PD value below 1.2 indicates a narrow dispersion. Thus, the polymer had a relatively uniform dispersion.

**Table 1:** Molecular weight determination of IOP.

RT (min)	lgMp	lgMw	lgMn	Mp	Mw	Mn	Peak area (%)
35.823	3.7	3.7	3.7	4,489	4,828	4,549	100

### 3.3. Nuclear magnetic resonance of *Inonotus obliquus* polysaccharide

**Figure 3:**
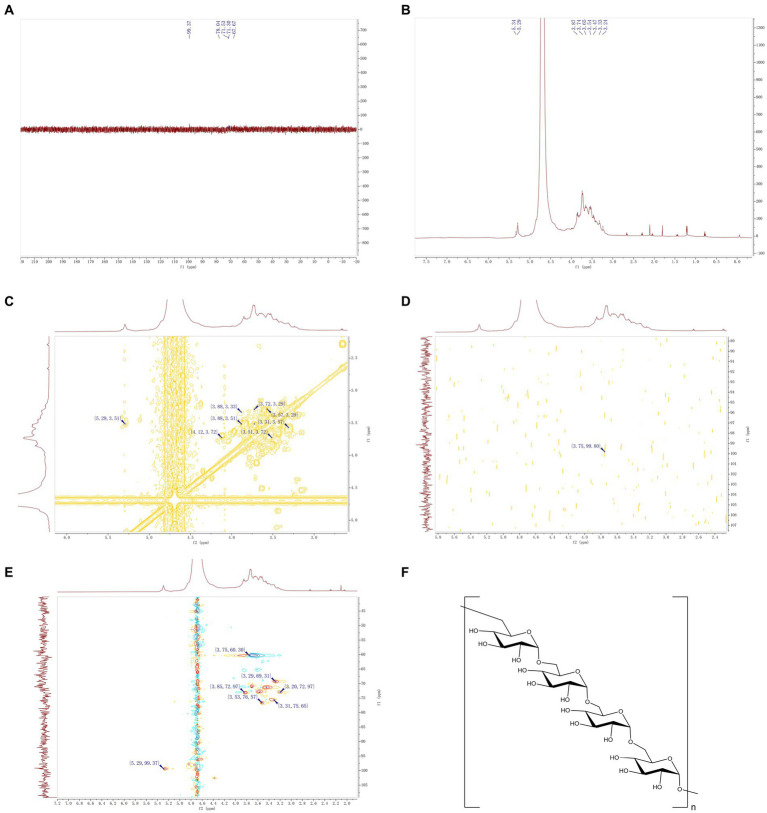
NMR of IOP. **(A)** Carbon spectrum of IOP, which represents the intramolecular carbon signal. **(B)** Hydrogen spectrum of IOP, which represents the intramolecular proton signal. **(C)** H-HCOSY 2D NMR spectrum of the IOP, representing the hydrogen-related signal on the adjacent carbon. **(D)** HMBC 2D NMR map of the IOP, representing a C–H related signal separated by 2–3 bonds. **(E)** HSQC 2D NMR spectrum of the IOP, representing the correlation signal between adjacent C–H molecules within the molecule. **(F)** Schematic representation of the molecular structure of the IOP, representing the way in which the residual sugars within the backbone of the polysaccharide are linked.

In the present study, the IOP structure was analyzed based on 1D (^1^H and ^13^C) NMR and 2D (HSQC, HMBC, COSY) NMR spectra. The ^13^C NMR and HSQC results (Figures 3A,C) revealed no obvious chemical shift signal of uronic acid within *δ*160–190 ppm, which demonstrated that IOP was a neutral substance. From the hydrogen spectrum of NMR (Figure 3B), it was found that the ^1^H NMR spectrum of IOP had a significant hydrogen signal in the range of 4.3–6.0 abnormal hydrogen, which is at *δ*5.29 ppm. Based on the NMR spectrum analysis of the polysaccharide material, the 2D HSQC (Figure 3C) revealed residual sugar in α configuration, which was confirmed by correlation signal with anechoic carbon at 5.29/99.37. From the overall analysis of the hydrogen and carbon spectra, the main IOP structure is composed of a six-carbon pyopyrum residual sugar of α configuration. Considering the extremely weak signal at other positions, the nuclear magnetic situation of the residual sugar of 5.29 ppm hetero-hydrogen, which is the majority, is discussed here. Preliminary findings showed that the signal is generated by the Glc glucose residue.

The chemical shifts at each position of the above major monosaccharide residues were identified by analyzing the 2D NMR spectra of HSQC (Figure 3C), HMBC (Figure 3D), and ^1^H-^1^H COSY (Figure 3E) in combination with the ^1^H and ^13^C NMR spectra. It is worth noting that the chemical shift of the anomer hydrogen and anomer carbon signals of the monosaccharide glucose residues is *δ*5.29/99.37 ppm. In the ^1^H-^1^H COSY spectrum, a correlation signal was found at *δ*5.29/3.51 ppm, validated by the H1/H2 signal in the residual sugar. The relevant information of *δ*3.53/76.57 ppm can be found (Figure 3E). The C2 chemical shift of this monosaccharide residue was 76.57 ppm. Using the same method, the chemical shift of H3 related to H2 was determined by ^1^H-^1^H COSY spectrum to be 3.51/3.72 ppm (Figure 3C), and the chemical shift of C3 corresponding to H3 in HSQC was found to be 71.30 ppm. The chemical shifts of H4/C4, H5/C5 and H6/C6 of the main monosaccharide residues were 3.29/69.31, 3.56/72.97 and 3.75/60.30, respectively. According to the HMBC spectrum (Figure 3D), there was a strong interference signal (3.75/99.80) at the position 99.37 of abnormal carbon and H-6 (3.75 ppm), which confirmed that the connection between the abnormal position of glucose and the C-6 position of IOP residue was the main component of IOP. The above results confirmed that the backbone of the IOP molecule is a linear structure composed of α-D-type glucose connected by the 1 → 6 position. The structure of the IOP backbone is shown in Figure 3F.

### 3.4. Cell experiment

**Figure 4:**
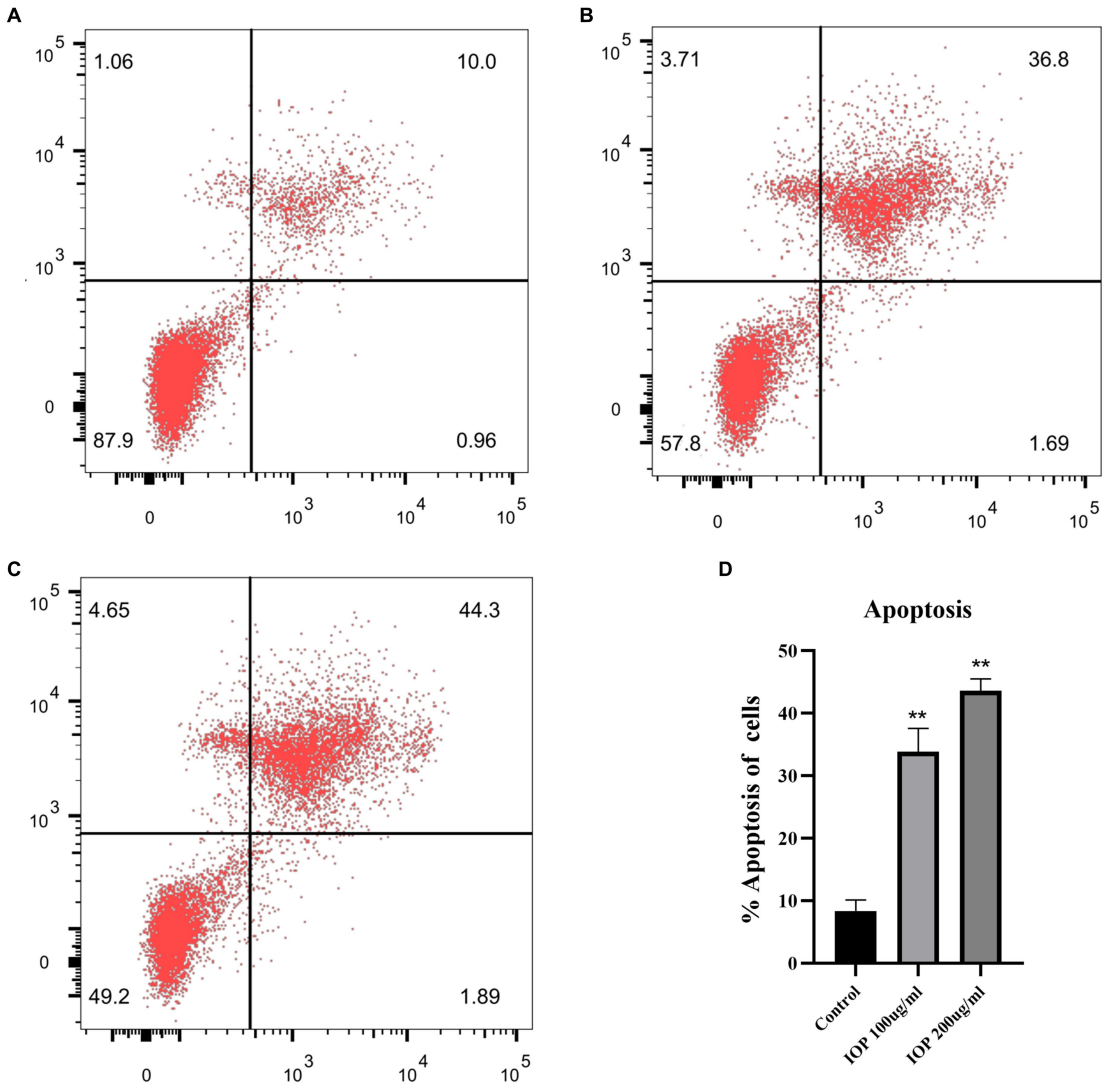
Effect of IOP on the apoptosis of LS411N cell line. **(A)** The apoptosis rate of LS411N in the control group (10.96%). **(B)** The apoptosis rate of LS411N cells treated with 100 μg/mL IOP (38.48%). **(C)** The apoptosis rate of LS411N cells treated with 200 μg/mL IOP (46.19%). **(D)** The data is presented as mean ± SD (*n* = 3). ^**^Represents *p* < 0.01. The comparisons were made between the experimental and the control group.

The effect of IOP on the apoptosis of LS411N cells was determined by flow cytometry (Figure 4). IOP increased the apoptosis rate of LS411N cells (Figure 4D, *p* < 0.01) in a concentration-dependent manner (Figure 4A for control: 10.96%; Figure 4B for 100 μg/mL IOP: 38.49%; Figure 4C for 200 μg/mL IOP: 46.19%). These results indicate that a low dose of IOP is not toxic to the body. At the same time, IOP can promote the apoptosis of cecum cancer cells (LS411N cells) in a dose-dependent manner.

### 3.5. Sex hormone levels

**Figure 5:**
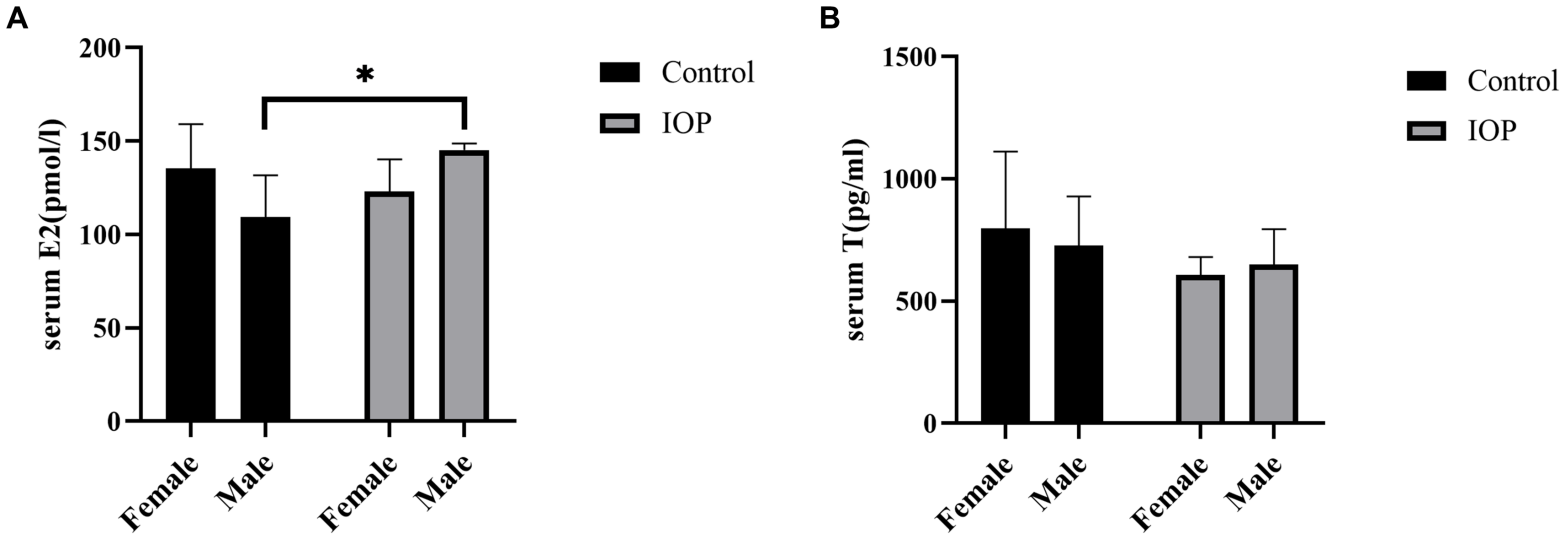
The effect of IOP on the secretion of sex hormone (*n* = 3). **(A)** E2 concentration in serum for four groups (pmol/L), ^*^p < 0.05. **(B)** T concentration in serum (pg/mL) following gavage IOP intake.

IOP treatment decreased the secretion of E2 in female rats but significantly increased the secretion of this hormone in male rats (Figure 5A). Meanwhile, IOP treatment decreased the secretion of T in both female and male rats, though not significantly (Figure 5B).

### 3.6. Effect of IOP on the overall structure of the gut microbiome

**Figure 6:**
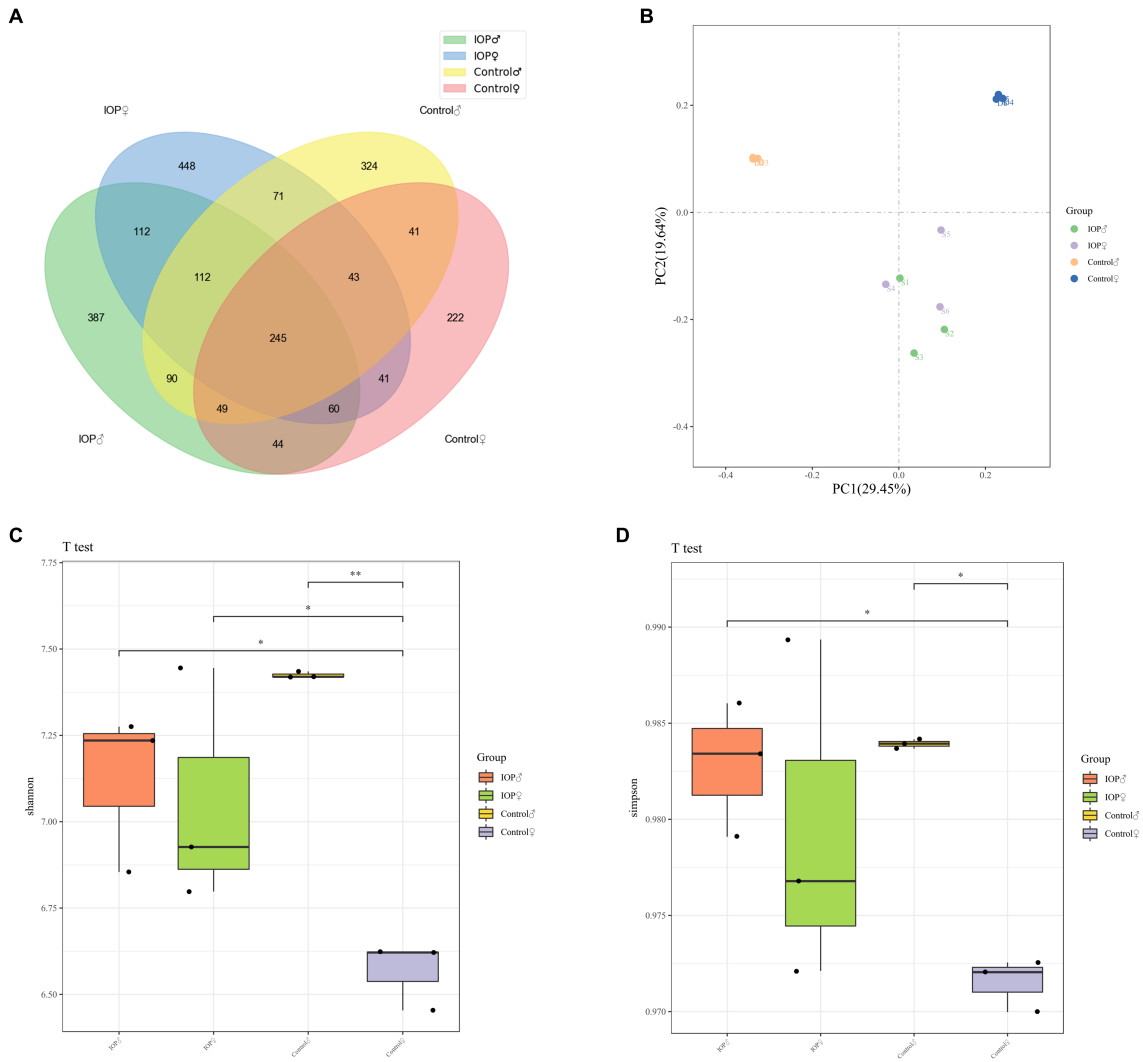
The effect of IOP on the overall structure of the gut microbiome. **(A)** Venn diagram indicates the number of common, unique ASVs between groups. The total ASV of the four groups is 245. **(B)** β diversity analysis. Unweighted UniFrac PCoA two-dimensional figure. The distance matrix algorithm was used to analyze the similarity between groups. The composition of intestinal microbiota was significantly different between IOP and control groups. However, the composition and abundance of cecal microbiota were similar between male and female rats after IOP intake. **(C)** Four groups of alpha Shannon indices, species diversity and species distribution. The diversity of caeca microbiota was significantly higher in male than in female rats in the control group, but there was no significant difference between male and female rats in the IOP group. The diversity of cecal flora increased significantly in female rats but not in male rats after IOP intake. ^*^p < 0.05, ^**^p < 0.01. **(D)** The α Simpson index. A higher Simpson index indicated higher community diversity. The diversity of caeca microbiota was significantly higher than that of female rats in the control group. However, no significant difference between male and female rats in the IOP group. The diversity of cecal flora increased significantly in female rats but not in male rats after IOP application. ^*^p < 0.05.

The raw data obtained from high-throughput sequencing ranged from 78,105 to 80,586, and the volume of valid tag data after removing chimeras was distributed between 466 and 671. Each group shared 245 ASVs (Figure 6A). The degree of diversity in each group was analyzed using α diversity, whereas the Shannon and Simpson indices were measured using the *t*-test. The Shannon and Simpson index results showed that the α diversity of males and females in the control groups was significantly different. The Shannon index of females in control and IOP groups was also significantly different. The results generally indicated that the diversity of caecal flora was higher in male than in female rats. However, IOP intake abrogated the difference (Figures 6C,D). Furthermore, β diversity analysis using PCoA plots revealed significant differences in cecal microbiota composition between male and female rats in the control group. This indicates that a shift was observed in the cecal microbiota after IOP intake. Generally, the caecal flora of male and female rats has a certain similarity (Figure 6B).

### 3.7. Effects of IOP on intestinal microbiota in rats

**Figure 7:**
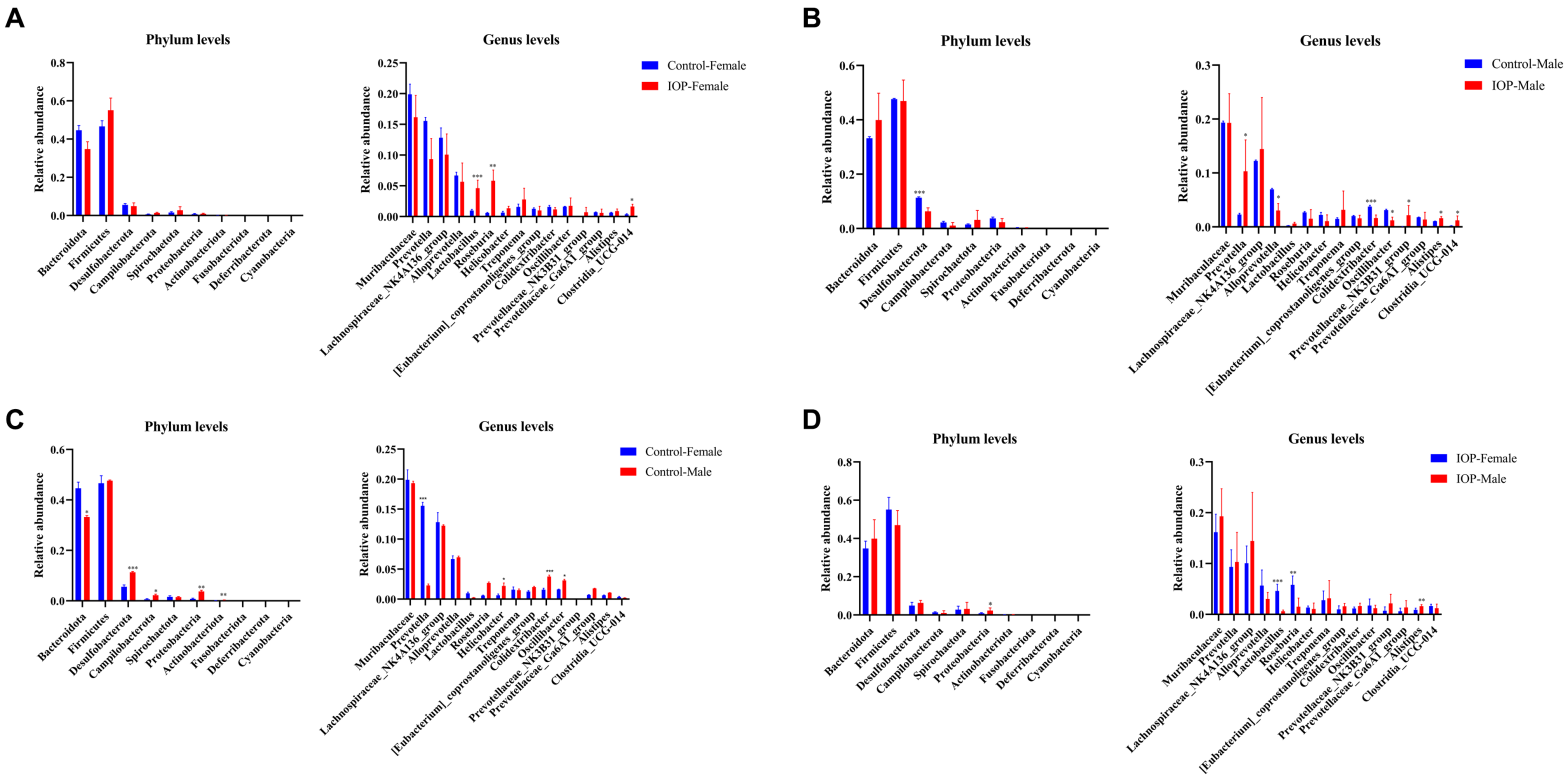
The effect of IOP on gut microbiota at the phyla level. **(A)** The abundance and composition of microbiota in the cecum of female rats after 100 mg/kg IOP intake. **(B)** The abundance and composition of microbiota in the cecum of male rats after 100 mg/kg IOP intake. **(C)** The difference in the abundance and composition of microbiota in cecum between female and male rats in the control group. **(D)** The difference in the abundance and composition of microbiota in the cecal between female and male IOP rats. ^*^p < 0.05, ^**^p < 0.01, and ^***^p < 0.001.

IOP intake significantly increased the abundance of members in the *Lactobacillus* (*p* < 0.001), *Roseburia* (*p* < 0.01), and *Clostridia_UCG-014* (*p* < 0.05) genera in female rat. However, no significant differences were observed at the phylum level in the female rats (Figure 7A). In contrast, at the phylum level, the abundance of *Desulfobacterota* (*p* < 0.001) decreased significantly in male rats. The abundance of members in the *Prevotella* (*p* < 0.05), *Prevotellaceae_NK3B31_group* (*p* < 0.05), *Alistipes* (*p* < 0.05) and *Clostridia_UCG-014* (*p* < 0.05) genus increased significantly in male rats. However, the abundance of *Alloprevotella* (*p* < 0.05), *Colidextribacter* (*p* < 0.001), and *Oscillibacter* (*p* < 0.05) decreased significantly (Figure 7B).

### 3.8. Difference in the abundance of intestinal microbiota in rats between sexes

At the phylum level, the abundance of *Desulfobacterota* (*p* < 0.001), *Campilobacterota* (*p* < 0.05), *Proteobacteria* (*p* < 0.01), and *Actinobacteriota* (*p* < 0.01) was significantly higher in male than female rats in the control group. The abundance of *Bacteroidota* (*p* < 0.05) was significantly lower in male rats than in female rats. At the genus level top15, the abundance of members in *Helicobacter* (*p* < 0.05), *Colidextribacter* (*p* < 0.001), and *Oscillibacter* (*p* < 0.05) general was significantly higher in male than in female rats. The abundance of *Prevotella* (*p* < 0.001) was significantly lower in male than in female rats (Figure 7C).

In the IOP group, the abundance of *Proteobacteria* (*p* < 0.05) was significantly higher in the top 10 male rats at the phylum level than in the female rats. In the top15 genus level, the abundance of *Alistipes* (*p* < 0.01) was significantly higher than in the female rats, while the abundance of *Lactobacillus* (*p* < 0.001) and *Roseburia* (*p* < 0.01) was significantly lower than in the female rats (Figure 7D).

**Figure 8:**
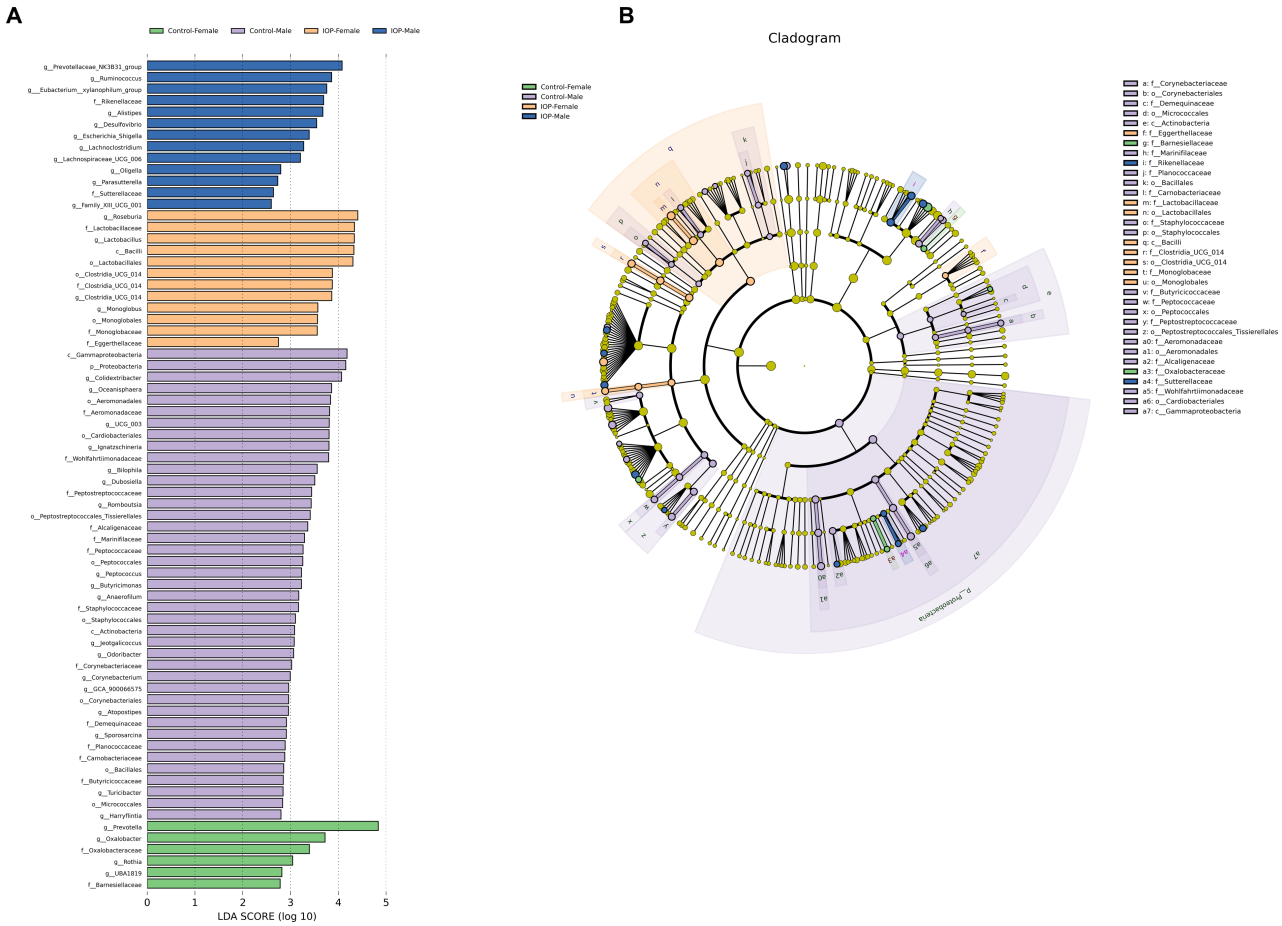
Linear discriminant analysis effect size (LEfSe) was used to analyze the differences in cecal microbiota among the control-female, control-male, IOP-female and IOP-male. **(A)** Bar graph of LDA distribution showing species at significant abundance (LDA >2) in different groups. The length of the bar graph represents the influence of significantly different species. **(B)** Evolutionary branch map for the control-female, control-male, IOP-female and IOP-male. The circles radiating from the inside represent taxonomic levels from phylum to genus (or species). Each small circle at a different taxonomic level represents a classification at that level, and the circle diameter length is proportional to the relative abundance size of the species.

LEfSe analysis showed that the abundance and composition of 72 bacterial species were significantly different among the four groups. The dominant bacteria in the control-female group were *Prevotella, Oxalobacter*, etc. The dominant bacteria in control-male group were *Proteobacteria, Colidextribacter, Oceanisphaera, Lgnatzschineria, Bilophila, Dubosiella, Romboutsia,* and *Peptococcus*, *Butyricimonas, Anaerofilum, Jeotgalicoccus, Odoribacter, Corynebacterium, Atopostipes, Sporosarcina, Turicibacter,* and *Harryflintia* among others. The dominant bacteria in the IOP-female group were *Roseburia, Lactobacillus* and *Clostridia_UCG_014*. The dominant bacteria in the IOP-male group were *Prevotellaceae_NK3B31_group*, *Ruminococcus*, *Eubacterium_xylanophilum_group*, *Alistipes, Desulfovibrio* and *Escherichia a_Shigella, Lachnoclostridium*, *Lachnospiraceae_UCG_006*, *Oligella,* and *Parasutterella* among others (Figure 8).

## 4. Discussion

Natural plant polysaccharides have attracted much attention increasing attention due to their diverse biological activities. The biological activities of most polysaccharides extracted from Chinese herbal medicine depend on their molecular structure, and monosaccharide compositions, structural diversity and molecular weight can all contribute to their varied biological properties (8). Studies have shown that polysaccharides that can protect the intestinal barrier are mostly composed of Gal, Man, Ara, Xly, and Rha (34–36). Our previous analysis using HPLC revealed that IOP is composed of Man, Glu A, Glu, Gal, Xyl, Ara and Fuc. Molecular weight is considered an important characteristic that affects the functionality of polysaccharides. It is generally believed that the molecular weight impacts the antioxidant activity, lipid-lowering, and antiviral properties of polysaccharides. Studies have shown that polysaccharides with lower molecular weight have better antioxidant activity (37), while polysaccharides with high molecular weight often preserve the gut barrier functions (38). In our study, we found that the peak molecular weight (Mp) of IOP was 4,489 Da, the number average molecular weight (Mn) was 4,549 Da, the weight average molecular weight (Mw) was 4,828 Da, and the polydispersity coefficient (PD) was 1.061. This indicates that IOP is a polysaccharide with a relatively low molecular weight. The symmetrical peaks in the HPGPC chromatogram and the total sugar content of 96.2% suggest that IOP is a pure polysaccharide with a homogeneous structure.

Polysaccharides composed of α-glycosidic linkages are less active than those composed of β-glycosidic linkages (39, 40). However, α-glucan can maintain intestinal homeostasis (41). We found that IOP has a linear structure composed of α-D-type glucose at the 1 → 6 position, and most glucose-lowering polysaccharides have a (1 → 6) glycosidic bond (42), indicating that IOP can protect the intestinal barrier including improving the permeability of intestinal barrier, maintaining intestinal homeostasis, and reduce blood glucose. Moreover, many plant polysaccharides have been shown to inhibit the growth of cancer cells. For instance, *sea mustard* polysaccharide (43) and *Lycium barbarum* polysaccharide (44, 45) inhibit the proliferation of cancer cells in a concentration-dependent manner, consistent with our results in which IOP induced the apoptosis of LS411N cell line. Notably, most polysaccharides can increase the abundance of beneficial bacteria in the gut. For example, *Astragalus* polysaccharides could improve the proliferation of beneficial bacteria, such as *Lactobacillus* and *Roseburia*, thus alleviating ulcerative colitis (46). Polysaccharide secreted by various mushroom species increases the abundance of *Prevotella* (47), consistent with our findings.

In the present study, IOP intake increased the abundance of *Lactobacillus*, *Alistipes, Roseburia, Prevotellaceae_NK3B31_group* and *Clostridia_UCG-014*, but reduced that of *Desulfobacterota, Colidextribacter*, *Oscillibacter* and *Alloprevotella* in rat cecum. *Alistipes* is a SCfas-producing bacteria, and the increase in abundance of *Alistipes* is related to the decrease in triglyceride (TG) concentration (48). Previous studies have shown that IOP can reduce obesity and the risk of developing other diseases by reducing the TG content (49). The abundance of *Prevotellaceae_NK3B31_group* was very low in the cecal of both male and female rats in the control group but increased significantly after IOP intake, especially in male rats. *Prevotellaceae_NK3B31* is a beneficial microorganism that promotes metabolism to alleviate diabetes by producing SCFAs (50–52). SCFAs maintain immune balance and not only play an important role in maintaining intestinal function but impact insulin secretion and reduce obesity (53). However, some polysaccharides cannot be directly digested in animals and need to be converted into SCFAs by intestinal microbiota to be absorbed by the body. Accordingly, these two microbiotas may contribute to IOP absorption by converting non-digestible matter in ruminants to SCFAs.

In addition, *Clostridia_UCG-014*, as a beneficial bacterium, promotes the digestion and absorption of nutrients (54). Furthermore, although *Colidextribacter* can convert polysaccharides to SCFAs, it also promotes the development of tumors by increasing the expression of oncogenes such as Bcl-2 (55, 56). *Desulfobacterota* is more abundant in rats with depression, and its abundance positively correlates with body weight and serum lipid level (17, 57). *Desulfobacterota* and *Colidextribacter* abundance decreased after IOP intake, which may be related to the improvement in the intestinal barrier and maintenance of microbial balance by IOP (58). The increase in the abundance of *Prevotellaceae_NK3B31_group* and *Alistipes* increased the content of SCFAs. *Clostridia_UCG-014* also promotes the absorption of IOP, increases the production of SCFAs, and balances the immune balances. A decrease in the abundance of *Desulfobacterota* and *Colidextribacter* reduces the risk of disease, such as depression and obesity, etc. (17, 57).

LEfSe analysis revealed a significant difference in the dominant bacteria in the cecum of female and male rats. In the control group, the abundance of *Actinobacteriota* was significantly higher in males than that in female rats, while *Bacteroidota* abundance was significantly lower in males than that in female rats. The abundance of *Bacteroidota* is thought to be associated with gender and body index (BMI) (59), while *Actinobacteriota* is positively correlated with body weight (60). After IOP intake, the dominant bacteria in the cecum of female and male rats changed. Notably, after IOP was applied, the F/B ratio changed from being higher in males than females to being higher in females than males. However, a recent study found that F/B was significantly higher in estrogen deficiency SD rat (61), consistent with our results. Sex-specific gut microbiota composition inherently differs between males and females of the same species, comparable with UniFrac PCoA index and alpha diversity analysis results in the present study.

At the same time, we also observed higher levels of E2 and T in female to male rats in the control group. Conversely, male rats exhibited higher levels of E2 and T than in female rats after IOP intake. Some researchers hypothesize that sex hormones may serve as the primary influencing the composition of intestinal flora across different genders (22). Furthermore, in healthy men, E2 levels are negatively correlated with alpha diversity (62), which may be due to the fact that E2 can alter the permeability of large intestines, facilitating the migration of gut microbiota (28, 63). In addition, the composition of gut microbiota, especially in the cecum, is strongly correlated with androgen production (both DHT and T) (64). For example, the abundance of *Prevotella* is positively correlated with T levels (65). In the present study the abundance of *Prevotella* increased significantly in male rats after IOP intake. *Roseburia*, which increased significantly in female rats, metabolizes butyrate to reduce gut PH (66), and the difference in pH is one of the reasons for the difference in the abundance and diversity of microbiota between sexes in the same species (13). Furthermore, studies have found that pectin can alter the composition of gut microbiota, which might reduce the abundance of species that improve gut immune function (67). Additionally, pH can alter the structure and activity of polysaccharides (68). Therefore, we speculate that the differences in the caecal microbiota of male and female rats caused by IOP may be attributed to structural changes of IOP under different pH environments. These findings create a foundation for further investigation into the specific functional properties of IOP’s structure.

## 5. Conclusion

The present study revealed the structure of IOP and the difference in the abundance and diversity in cecal microbiota between male and female rats after IOP intake. IOP has a linear backbone composed of an α-D-type glucose chain connected through the 1 → 6 position. At the same time, IOP is beneficial in the body as it not only induces apoptosis of LS411N cells but also increases the abundance of some beneficial bacteria, including *Prevotellaceae_NK3B31_group* and *Clostridia_UCG-014*. These bacteria play a significant role in the digestion and absorption of polysaccharides in the body. In addition, it reduced the abundance of some harmful bacteria, such as *Desulfobacterota* and *Colidextribacter*, reducing the risk of certain diseases. In addition, our study also showed that differences exist in the diversity of caecal microbiota between female and male rats, which was abrogated by IOP intake. The sex-induced changes in dominant bacteria may be related to sex hormone levels, and the changes in male rats are more stable than those in female rats, but the specific reason for this phenomenon requires further investigation.

## Data availability statement

The datasets presented in this study can be found in online repositories. The names of the repository/repositories and accession number(s) can be found in the article/supplementary material.

## Ethics statement

The animal study was approved by The Animal Care Office of Chengdu Normal University (No: CDNU-2021092614M). The study was conducted in accordance with the local legislation and institutional requirements.

## Author contributions

SL, JZ, and BH: data curation and writing—review and editing. WZ, YW, and XD: formal analysis and methodology. SL: funding acquisition. XD, WZ, and BH: investigation. WZ, WJ, and XD: software. BH and JZ: validation. WZ and SL: writing—original draft. All authors contributed to the article and approved the submitted version.
